# Deriving objectively-measured sedentary indices from free-living accelerometry data in rural and urban African settings: a cost effective approach

**DOI:** 10.1186/s13104-019-4606-4

**Published:** 2019-09-12

**Authors:** Ian Cook

**Affiliations:** Physical Activity Epidemiology Laboratory, Faculty of Humanities, University of Limpopo (Turfloop Campus), P.O. Box 459, Fauna Park, Polokwane, Limpopo Province 0787 South Africa

**Keywords:** Sedentarism, Accelerometer, Movement monitor, Measurement

## Abstract

**Objectives:**

To investigate the agreement between two data reduction approaches for detecting sedentary breaks from uni-axial accelerometry data collected in human participants. Free-living, uni-axial accelerometer data (n = 318) were examined for sedentary breaks using two different methods (Healy–Matthews; MAH/UFFE). The data were cleaned and reduced using MAH/UFFE Analyzer software and custom Microsoft Excel macro’s, such that the average daily sedentary break number were calculated for each data record, for both methods.

**Results:**

The Healy–Matthews and MAH/UFFE average daily break number correlated closely (R^2^ = 99.9%) and there was high agreement (mean difference: + 0.7 breaks/day; 95% limits of agreement: − 0.06 to + 1.4 breaks/day). A slight bias of approximately + 1 break/day for the MAH/UFFE Analyzer was evident for both the regression and agreement analyses. At a group level there were no statistically or practically significant differences within sample groups between the two methods.

## Introduction

The seminal study of Healy et al. demonstrated the usefulness of objectively-measured indices of sedentary behaviour, namely sedentary time and specifically sedentary breaks (periods of physical activity punctuating sedentary time) [1]. Subsequent to the Healy et al. study, further evidence has emerged supporting the use of objectively-measured sedentary time and sedentary breaks [2–4]. More recently, in addition to sedentary time and breaks, the importance of sedentary bout durations has been highlighted [5]. The accumulating evidence regarding the importance of sedentary behaviour [6, 7] has led to a proliferation of terminology, definitions, measurement techniques and methods [3, 8–10].

To date, the extraction of these objectively-measured sedentary-related variables, has utilized software which requires some level of programming skills (R, SAS, MatLab) [1–3, 5, 9]. More recently, commercial proprietary software such as Actilife [4] and MeterPlus™ [11] provide researchers with the ability to extract these variables without the need for programming skills. However, this software is costly such that an additional barrier exists for researchers from low resource settings to implement these variables in their analyses. Initial software costs range from approximately USD 695 to USD 1695 for a single-user licence, and an additional USD 200 annual renewal per licence.

Freeware software such as MAH/UFFE has been used extensively to clean and score accelerometry data whether describing physical activity (non-sedentary time) (≥ 100 counts) or sedentary time (< 100 counts). As of May 2019, approximately 329 items were retrieved from Google Scholar (excluding citations and patents) using the search term ‘MAHUFFE’, and 79 items were retrieved from PubMed Central (4 papers reported the use of MAHUFFE for 2017–2019).

However, it must be stated that a sedentary break is in essence the initiation of a bout of physical activity. Since a cut-point or threshold is employed (≥ 100 counts) [1], it is thus possible to specify parameters within the MAH/UFFE configuration such that sedentary breaks can be easily determined. Importantly, as far as the author is aware, no study has used MAH/UFFE to extract sedentary breaks. Consequently, this analysis evaluates the agreement in indentifying sedentary breaks using the accepted Healy–Matthews algorithm [1] and the MAH/UFFE accelerometer data reduction software, using accelerometer data collected in rural and urban African participants during a physical activity questionnaire validation study [12, 13] and a free-living, cross-sectional survey [14]. Previous analyses of this data did not include sedentary breaks as a sedentary index. Because the initial analysis of this data utilized MAH/UFFE, further analyses of this data will require the use of the same software to maintain consistency. Hence, there is a need to determine the validity of using MAH/UFFE to extract sedentary breaks against an accepted standard.

## Main text

### Methods

The data for this analysis comprises three samples (n = 318) and has been reported in detail elsewhere [12–14].

#### Dikgale Health and Demographic Surveillance System site (DHDSS) sample [14]

Rural, adult females resident in the DHDSS [15], were conveniently recruited (n = 262). The participants generally performed subsistence tasks (housework, fetching wood and water, walking as a means of transport).

#### Rural sample sample [12, 13]

Male and female adults, resident on farms and villages, were conveniently recruited from a local lumber mill situated in the Limpopo Province, South Africa (n = 30). These participants performed a variety of manual tasks (plantations created and maintained, raw timber harvested, sized, cleaned and stacked).

#### Urban sample sample [12, 13]

A convenience sample was recruited from male and female adult staff and students of the University of the Limpopo (Turfloop Campus), and adult residents (office workers, teachers) from the surrounding community (Mankweng) and nearby city (Polokwane) (n = 26). The participants performed tasks typical of office workers (sitting, standing quietly and bouts of exercise; gymnasium, walking/jogging, sport).

#### Data collection and initial data reduction

The initial data reduction methodology is described in detail elsewhere [14]. In short, participants were asked to wear uni-axial accelerometers for six to seven complete days. The CSA model 7164 (Rural and Urban sample) and MTI model AM-7164-2.2 (DHDSS sample) are both products of Actigraph, LLC, Pensacola, FL, USA (formerly Computer Science Applications, Inc. Shalimar, FL and MTI Health Services, Fort Walton Beach, FL). The minute-by-minute data were downloaded from the accelerometers onto an IBM-compatible personal computer via an interface unit, for further analysis using specialized software (MAH/UFFE Analyzer version 1.9.0.3; http://www.mrc-epid.cam.ac.uk/physical-activity-downloads/). Unlike an earlier analysis [14], the MAH/UFFE configuration file was modified such that sedentary breaks of ≥ 1 min could be detected and summarized (see Additional file 1). In this case, the MAH/UFFE settings detect ≥ 1 min bouts of ≥ 100 acceleration counts. In other words, the number of sedentary breaks and total break time are determined. The resulting summary Microsoft Excel file was imported into a statistical package for further analysis. From the total number of sedentary breaks and total break time, a daily average was calculated for each participant by dividing the totals by the number of valid days.

#### Additional data reduction and sedentary break analysis

**Fig. 1:**
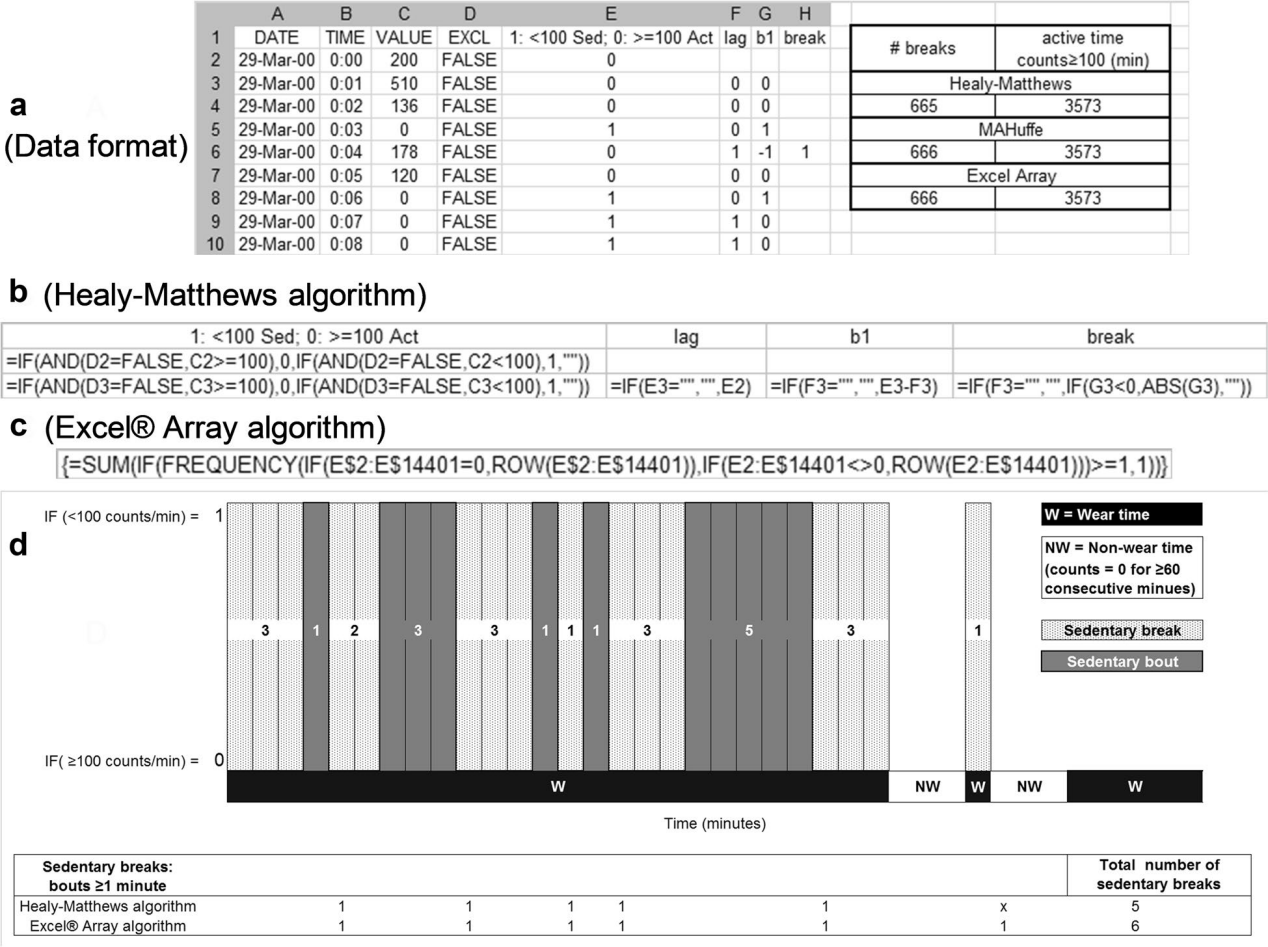
Diagrammatic representation of the data structure and algorithms. **a** Data format of an individual, cleaned Excel data file (column A–D) including the Healy–Matthews algorithm (column E–H); **b** cell functions for the Healy–Matthews algorithm; **c** cell content and function for a Microsoft Excel Array algorithm; **d** sedentary break identification for two algorithms

For this analysis individual, minute-by-minute data files (CSV, long format; Fig. 1a) created with MAH/UFFE were batch-converted to individual Microsoft Excel files using a custom Microsoft Visual Basic macro. Thereafter, the data for non-valid days (indentified in the initial data reduction) and non-wear time (identified in the initial data reduction) were removed for each individual, minute-by-minute Microsoft Excel file using a customized Microsoft Visual Basic macro. Finally, the number of total sedentary breaks and total break time for the Healy–Matthews algorithm [1] and a Microsoft Excel Array function were calculated for each cleaned, individual minute-by-minute Microsoft Excel file using a customized Microsoft Visual Basic macro. The resulting summary Microsoft Excel file was imported into a statistical package for further analysis. From the total number of sedentary breaks and total break time, a daily average was calculated for each participant by dividing the totals by the number of valid days. The Microsoft Visual Basic macros used in this analysis can be obtained from the author by request.

The SAS syntax for the Healy–Matthews algorithm [1] was obtained from the authors (Dr Genevieve N Healy, personal communication) and implemented within a Microsoft Excel spreadsheet (Fig. 1a, b). Unlike the Healy–Matthews algorithm, the MAH/UFFE software does not require the deletion of excluded values (non-wear time, counts = 0 for 60 consecutive minutes) when detecting bouts, but rather codes the excluded values as “TRUE” (Fig. 1a, Column D). Counts ≤ 99 were defined as sedentary and coded as “1” (Fig. 1a, Column E). The Healy–Matthews algorithm requires the use of a “lag” function to identify a sedentary break (Fig. 1a, Column F). The total break time (counts ≥ 100) was calculated by counting the number of zeros (Fig. 1a, Column E).

In addition, a Microsoft Excel Array function (Fig. 1c) was used to detect the number of sedentary breaks or the number of one-or-more consecutive zero’s (Fig. 1a, Column E). The total break time (counts ≥ 100) was calculated using a Microsoft Excel Array function (Fig. 1a, Column E).

Figure 1d is a diagrammatic representation of the first 26 min of a participant’s data in Fig. 1a, and also illustrates a potential sedentary break (activity) bracketed by two non-wear time periods. In this example there are 11 min of sedentary behaviour (counts ≤ 99) distributed as 5 continuous sedentary bouts, and 5 corresponding sedentary breaks, within the first 26 min.

Descriptive statistics comprised means and one standard deviation (SD). One-way Analysis of Variance was used to compare variables across groups (samples and algorithms). Post hoc multiple comparison analyses (Sidak’s t-test) assessed group differences. Linear regression was used to examine the relationship between output variables from the Healy–Matthews and MAH/UFFE algorithms. Bland–Altman plots explored the agreement between the outputs of the Healy–Matthews and MAH/UFFE algorithms. Data were analysed using appropriate statistical software (Stata/SE for Windows: Release 15.1. College Station, TX: StataCorp LP, 2018). Significance was set at *p *< 0.05.

### Results

Considering the sedentary breaks per day and the minutes per break, across the three samples, and the Healy–Matthews and MAH/UFFE algorithms, there was no statistical or practical difference between the algorithms (Table 1).

**Table 1:** General characteristics and accelerometer indices across participant groups, and between algorithms for sedentary indices

	Urban (office, n = 26)	Rural (forestry, n = 30)	Rural (DHDSS, n = 262)
Female (%)^a^	50	40	100
Age (years)	31.8 (6.6)	36.6 (10.1)	35.1 (10.5)
Body mass index (kg m^−2^)	26.9 (5.6)	22.3 (3.5)^b^	26.9 (5.6)
Days monitored	5.0 (1.4)	5.7 (1.3)	5.5 (1.6)
Registered time (min day^−1^)	860 (64)	974 (163)^b^	870 (140)
Active time (min day^−1^)	365 (65)	545 (75)^b^	422 (83)^c^
Average active counts (cts min^−1^)	973 (310)	1256 (286)^b^	956 (195)
Light activity (min day^−1^)	223 (43)	248 (58)^d^	218 (50)
Moderate-1 activity (min day^−1^)	86 (24)^e^	187 (44)^e^	157 (53)^e^
Moderate-2 to Vigorous activity (min day^−1^)	56 (33)	110 (50)^b^	47 (29)
Sedentary time (min day^−1^)	1075 (65)	896 (75)^b^	1018 (83)^c^
Sedentary breaks (breaks ≥ 1 min)			
Number per day			
MAH/UFFE algorithm	87 (11)^f^	76 (10)	71 (14)
Healy–Matthews algorithm	86 (11)	75 (10)	70 (14)
Minutes per break			
MAH/UFFE algorithm	4.2 (0.8)^e^	7.3 (1.4)^e^	6.2 (1.8)^e^
Healy–Matthews algorithm	4.3 (0.8)	7.3 (1.4)	6.2 (1.8)
Total counts (cts day^−1^)	366 862 (136 960)	692 972 (193 778)^b^	412 776 (124 832)
Average counts (cts day^−1^ min^−1^)	432 (171)	721 (193)^b^	482 (155)

*DHDSS* Dikgale Health and Demographic Surveillance System Site

Except ^a^values are reported as raw mean (sd); Active: ≥ 100 cts min^−1^; Light: 100–759 cts min^−1^; Moderate-1: 760–1951 cts min^−1^; Moderate-2 to Vigorous: ≥ 1952 cts min^−1^; Sedentary: ≤ 99 cts min^−1^; Average counts = Total counts/registered time

^b^Rural (Forestry) vs Urban (Office), Rural (DHDSS) *p *≤ 0.010

^c^Rural (DHDSS) vs Urban (Office) *p *< 0.0001

^d^Rural (Forestry) vs Rural (DHDSS) *p *= 0.007

^e^Rural (Forestry) vs Urban (Office) vs Rural (DHDSS) *p *≤ 0.007

^f^Urban (Office) vs Rural (Forestry), Rural (DHDSS) *p *≤ 0.005

Moreover, there were no significant differences across the algorithms for the combined data (n = 318) for both average breaks per day or average total break time (*p *≥ 0.911); the values were virtually identical across algorithms (average total break time: 429 ± 90 min day^−1^, average break number: 72 ± 14 to 73 ± 14 breaks day^−1^).

**Fig. 2:**
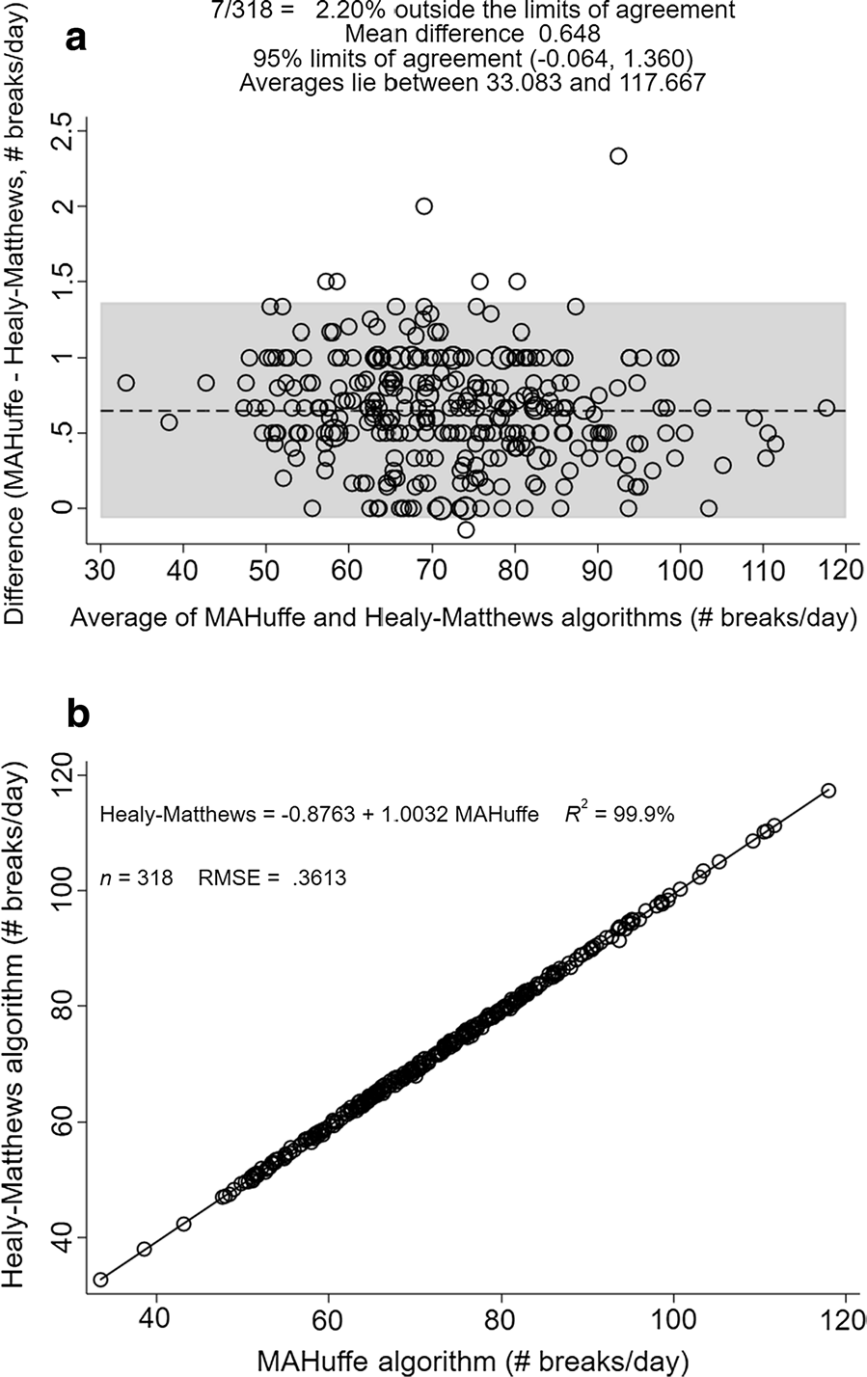
Agreement analyses between the outputs of the two algorithms. **a** Bland–Altman plot between the Healy–Matthews algorithm and the MAH/UFFE for average breaks/day; **b** Linear regression plot between the Healy–Matthews algorithm and MAH/UFFE for average breaks/day

Both the agreement analysis (Fig. 2a) and linear regression (Fig. 2b), demonstrated a slight bias toward detecting approximately 1 sedentary break per day more with the MAH/UFFE software. Only 2.2% of the data points fell outside the 95% limits of agreement; the maximum difference was < 2.5 sedentary breaks per day (Fig. 2a).

### Discussion

This analysis is novel in that, as far as the author is aware, this is the first time that it has been demonstrated that the MAH/UFFE data reduction software is capable of identifying sedentary breaks in high agreement with the accepted Healy–Matthews algorithm. In addition, since MAH/UFFE uses Microsoft Excel in its underlying architecture, it is not surprising that the Microsoft Excel Array functions agreed well with the MAH/UFFE output.

Second, this analysis provides novel sedentary data. As far as the author is aware, this is the first sedentary break data from a rural African setting. The number of breaks per day reported from more urbanized settings [2–5] tends to be higher than the values reported for the two rural groups in this study, but similar to the urban sample. Further analysis is required around the descriptive epidemiology of these indices in rural African rural samples, and the independent relationship of these sedentary indices to health variables.

The likely explanation for the slight bias is the manner in which non-wear periods are dealt with by the two algorithms. The Healy–Matthews algorithm [1] requires the physical removal of non-wear time, such that any sedentary break bracketed by non-wear time will most likely be added to an activity period on either side of the non-wear-time. However, it is possible that such a bracketed sedentary break could also remain as such, if the valid wear times, adjacent to the non-wear times, are valid sedentary bouts. On the other hand, the MAH/UFFE algorithm does not physically remove the non-wear time, and thus allows a sedentary break, bracketed as such, to be counted as a valid sedentary break i.e. a period of activity. In essence, the MAH/UFFE algorithm characterizes the non-wear times as sedentary. However, the agreement analysis suggests that although this does occur often (93% of differences > 0), 98% of the differences are ≤ 1.4 sedentary breaks per day. It seems unlikely that these algorithmic artefacts are of any practical significance, especially at the group level. This analysis does also demonstrate that the Healy–Matthews algorithm [1] can be employed on MAH/UFFE processed data files using Excel and custom macro’s, such that more direct comparisons can be made to published data [2–5], although, as stated earlier, it is unlikely that the differences will be practically significant when using the MAH/UFFE algorithm.

While this analysis has shown the utility of using an existing data reduction programme to identify important sedentary behaviour indices, MAH/UFFE is not capable of detecting bouts of sedentary time (≤ 99 counts) as suggested by Kim et al. [5]. A solution is to use Microsoft Excel Array functions to detect sedentary bouts.

It is also important to note that the MAH/UFFE data reduction software is only capable of reducing Actigraph uni-axial data files, not bi- or tri-axial Actigraph data files. However, a file converter is available which can convert bi- or tri-axial files to uni-axial or vector magnitude data files, which in turn can be reduced with the MAH/UFFE software.

This paper has shown that a ubiquitous measure in sedentary behaviour research, namely sedentary breaks, can be extracted with high agreement to an accepted standard, with minimal effort and cost.

## Limitations

Due to the cross-sectional, convenience sampling in this study, the results cannot be readily generalized to the respective rural and urban populations from whence the participants were recruited.

## Supplementary information

**Additional file 1.** MAH/UFFE Settings: settings required by the configuration file (MAHUFFE_1903.ini) for identifying Sedentary Breaks (bouts ≥ 1 min for counts ≥ 100).

## Data Availability

The data analysed during the current study are not publicly available due to the original consent and ethics approval not containing approval from the participants for data sharing. Reasonable requests would be considered in consultation with the University of Limpopo Ethics Committee and the various community leaders.

## Abbreviation

DHDSS: Dikgale Health and Demographic Surveillance System Site

## References

[1] Healy GN, Dunstan DW, Salmon J, Cerin E, Shaw JE, Zimmet PZ (2008). Breaks in sedentary time: beneficial associations with metabolic risk. Diabetes Care.

[2] Bankoski A, Harris TB, McClain JJ, Brychta RJ, Caserotti P, Chen KY (2011). Sedentary activity associated with Metabolic Syndrome independent of physical activity. Diabetes Care.

[3] Healy GN, Matthews CE, Dunstan DW, Winkler EAH, Owen N (2011). Sedentary time and cardio-metabolic biomarkers in US adults: NHANES 2003–06. Eur Heart J.

[4] Bellettiere J, Healy GN, LaMonte MJ, Kerr J, Evenson KR, Rillamas-Sun E (2019). Sedentary behavior and prevalent diabetes in 6,166 older women: the Objective Physical Activity and Cardiovascular Health Study. J Gerontol A Biol Sci Med Sci.

[5] Kim Y, Welk GJ, Braun SI, Kang M (2015). Extracting objective estimates of sedentary behavior from accelerometer data: measurement considerations for surveillance and research applications. PLoS ONE.

[6] Ford ES, Caspersen CJ (2012). Sedentary behaviour and cardiovascular disease: a review of prospective studies. Int J Epidemiol.

[7] Owen N, Bauman A, Brown W (2009). Too much sitting: a novel and important predictor of chronic disease risk?. Br J Sports Med.

[8] Atkin AJ, Gorely T, Clemes SA, Yates T, Edwardson C, Brage S (2012). Methods of measurement in epidemiology: sedentary behaviour. Int J Epidemiol.

[9] Keadle SK, Sampson JN, Li H, Lyden K, Matthews CE, Carroll RJ (2017). An evaluation of accelerometer-derived metrics to assess daily behavioral patterns. Med Sci Sports Exerc.

[10] Tremblay MS, Aubert S, Barnes JD, Saunders TJ, Carson V, Latimer-Cheung AE (2017). Sedentary behavior research network (SBRN)—terminology consensus project process and outcome. Int J Behav Nutr Phys Act..

[11] O’Connor SG, Habre R, Bastain TM, Toledo-Corral CM, Gilliland FD, Eckel SP (2019). Within-subject effects of environmental and social stressors on pre- and post-partum obesity-related biobehavioral responses in low-income Hispanic women: protocol of an intensive longitudinal study. BMC Public Health..

[12] Cook I, Lambert EV (2002). Validity and reliability of the International Physical Activity Questionnaire in Northern Sotho—speaking Africans. JEMDSA..

[13] Cook I, Lambert EV (2008). The sources of variance and reliability of objectively monitored physical activity in rural and urban Northern Sotho-speaking Africans. S Afr J Sports Med..

[14] Cook I, Alberts M, Lambert EV (2012). Influence of cut-points on patterns of accelerometry-measured free-living physical activity in rural and urban black South African women. J Phys Act Health..

[15] Alberts M, Dikotope SA, Choma SR, Masemola ML, Modjadji SE, Mashinya F (2015). Health and demographic surveillance system profile: the Dikgale Health and Demographic Surveillance System. Int J Epidemiol.

